# Identification of amino acid residues of nerve growth factor important for neurite outgrowth in human dorsal root ganglion neurons

**DOI:** 10.1111/ejn.14513

**Published:** 2019-08-01

**Authors:** Märta Dahlström, Gunnar Nordvall, Erik Sundström, Elisabet Åkesson, Gunilla Tegerstedt, Maria Eriksdotter, Pontus Forsell

**Affiliations:** ^1^ Division of Clinical Geriatrics Department of Neurobiology, Care Sciences and Society Karolinska Institutet Huddinge Sweden; ^2^ AlzeCure Foundation Huddinge Sweden; ^3^ Division of Neurogeriatrics Department of Neurobiology, Care Sciences and Society Karolinska Institutet Huddinge Sweden; ^4^ AlzeCure Pharma AB Huddinge Sweden; ^5^ R&D Unit Stockholms Sjukhem Stockholm Sweden; ^6^ Division of Gynecology and Obstetrics Department of Clinical Science, Intervention and Technology Karolinska Institutet Huddinge Sweden; ^7^ Theme Aging Karolinska University Hospital Stockholm Sweden

**Keywords:** human cell cultures, immunocytochemistry, nerve growth factor (NGF) mutants, TrkA signalling

## Abstract

Nerve growth factor (NGF) is an essential neurotrophic factor for the development and maintenance of the central and the peripheral nervous system. NGF deficiency in the basal forebrain precedes degeneration of basal forebrain cholinergic neurons in Alzheimer's disease, contributing to memory decline. NGF mediates neurotrophic support via its high‐affinity receptor, the tropomyosin‐related kinase A (TrkA) receptor, and mediates mitogenic and differentiation signals via the extracellular signal‐regulated protein kinases 1 and 2 (ERK1/2). However, the molecular mechanisms underlying the different NGF/TrkA/ERK signalling pathways are far from clear. In this study, we have investigated the role of human NGF and three NGF mutants, R100E, W99A and K95A/Q96A, their ability to activate TrkA or ERK1/2, and their ability to induce proliferation or differentiation in human foetal dorsal root ganglion (DRG) neurons or in PC12 cells. We show that the R100E mutant was significantly more potent than NGF itself to induce proliferation and differentiation, and significantly more potent in activation of ERK1/2 in DRG neurons. The W99A and K95A/Q96A mutants, on the other hand, were less effective than the wild‐type protein. An unexpected finding was the high efficacy of the K95A/Q96A mutant to activate TrkA and to induce differentiation of DRG neurons at elevated concentrations. These data demonstrate an NGF mutant with improved neurotrophic properties in primary human neuronal cells. The R100E mutant represents an interesting candidate for further drug development in Alzheimer's disease and other neurodegenerative disorders.

## Graphical abstract

The nerve growth factor (NGF) mutant R100E is more potent to induce proliferation, differentiation (anti‐β‐tubulin green staining) and phosphorylation of ERK1/2 (red staining) than wild‐type NGF in human foetal dorsal root ganglion (DRG) neurons after a 4‐day treatment. The NGF double mutant K95A/Q96A is more efficient to induce neuronal differentiation than wild‐type NGF in DRG neurons at high concentrations (>3 μg/ml). We show enhanced functional response in human nerve cells with NGF mutants.

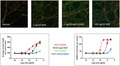

**Abbreviations**
AD: Alzheimer's disease
BDNF: Brain‐derived neurotrophic factor
BSA: Bovine serum albumin
cDNA: Complementary DNA
CNS: Central nervous system
DMEM: Dulbecco's modified Eagle's medium
DNA: Deoxyribonucleic acid
DRG: Dorsal root ganglion
EC_50_: Half‐maximal effective concentration
EDTA: Ethylenediaminetetraacetic acid
EFC: Enzyme fragment complementation
EK: Enterokinase
EMEM: Eagle's minimum essential medium
ERK1/2: Extracellular signal‐regulated protein kinases 1 and 2
FBS: Foetal bovine serum
GSH: Glutathione
GSSG: Glutathione disulphide
HSAN IV: Hereditary sensory and autonomic neuropathy type IV
HSAN V: Hereditary sensory and autonomic neuropathy type V
IPTG: Isopropyl β‐D‐1‐thiogalactopyranoside
LB: Lysogeny broth
MAPK: Mitogen‐activated protein kinase
NA: Not applicable
*NGFB*: Nerve growth factor beta gene
NGF: Nerve growth factor
NIR: Near‐infrared
NT3: Neurotrophin‐3
NT4/5: Neurotrophin‐4/5
*NTRK1*: Neurotrophic receptor tyrosine kinase 1 gene
p75: p75 neurotrophin receptor
PBS: Phosphate‐buffered saline
PC12: Rat pheochromocytoma PC12 cells
PDL: Poly‐D‐lysine
pERK1/2: phospho‐ERK1/2
PI3K: Phosphoinositide 3‐kinase
PLCγ1: Phospholipase C gamma 1
PNS: Peripheral nervous system
pTrkA: Phospho‐TrkA
PVDF: Polyvinylidene difluoride
rEK: Recombinant enterokinase
RIPA: Radioimmunoprecipitation assay
SDS‐PAGE: Sodium dodecyl sulphate–polyacrylamide gel electrophoresis
SEC: Size‐exclusion chromatography
SEM: Standard error of the mean
SHC1: Src homology 2 domain containing transforming protein 1
TBS: Tris‐buffered saline
TrkA: Tropomyosin‐related kinase A
U2OS: Human osteosarcoma U2OS cells

## 1 INTRODUCTION

Nerve growth factor (NGF) was first discovered by the Italian neurobiologist Rita Levi‐Montalcini in the mid‐20th century (Levi‐Montalcini, 1952). NGF is a member of the neurotrophin family that also includes brain‐derived neurotrophic factor (BDNF), neurotrophin‐3 (NT3) and neurotrophin‐4/5 (NT4/5) (Barde, Edgar, & Thoenen, 1982; Ernfors, Ibáñez, Ebendal, Olson, & Persson, 1990; Hallböök, Ibáñez, & Persson, 1991). Prenatally, NGF and other neurotrophins contribute to the differentiation, proliferation and survival of neurons and the formation of functional synapses in the nerve fibres, both in the central nervous system (CNS) and in the peripheral nervous system (PNS). In the adult, basal forebrain cholinergic neurons maintain their reliance on NGF (Hefti, 1986). Reduced levels of mature NGF available to the basal forebrain precede the loss of cholinergic neurons and the impaired cognitive functions observed in Alzheimer's disease (AD) (Alzheimer, Stelzmann, Schnitzlein, & Murtagh, 1995), thus making NGF an important contributor to neuronal survival (Hefti & Mash, 1989; Niewiadomska, Mietelska‐Porowska, & Mazurkiewicz, 2011) and useful as a therapeutic agent in AD (Cattaneo & Calissano, 2012; Iulita & Cuello, 2015). Administration of NGF directly into the basal forebrain indicates positive effects in AD patients with respect to biomarkers, histology and cognitive functions, and shows no side effects (Eriksdotter‐Jönhagen et al., 2012; Eyjolfsdottir et al., 2016; Ferreira et al., 2015; Karami et al., 2015; Tuszynski et al., 2005; Wahlberg et al., 2012). NGF binds to either the receptor tropomyosin‐related kinase A (TrkA) which enhances survival and differentiation or the p75 neurotrophin receptor (p75) resulting in pro‐apoptotic signalling.

The crystal structure of NGF itself as well as in complex with TrkA has been determined (He & Garcia, 2004; McDonald et al., 1991; Ultsch et al., 1999; Wiesmann, Ultsch, Bass, & de Vos, 1999). These studies have demonstrated that NGF forms a homodimer with three antiparallel pairs of β‐strands and four loop regions in each monomer. Notably, NGF loop 1 (amino acid residue 29–35) or loop 4 (amino acid residue 91–97) alone is sufficient to induce differentiation in terms of neurite outgrowth to the same extent as full‐length NGF (Gudasheva et al., 2015; Xie, Tisi, Yeo, & Longo, 2000). Crystal structure studies of NGF also revealed a binding pocket for lipids (Tong et al., 2012), where the lipid is anchored to NGF by interaction with several amino acid residues from both monomers of NGF, including W99. Binding of lysophosphatidylserine is important for the function of NGF in mast cells and regulates the release of histamine and serotonin (Tong et al., 2012). The lipid‐binding site is adjacent to the position of the amino acid R100, which plays an important role in hereditary sensory and autonomic neuropathy type V (HSAN V) (Minde et al., 2004). HSAN V is caused by a single point mutation in the nerve growth factor beta gene (*NGFB*) (Einarsdottir, 2004), leading to an amino acid substitution from arginine (R) to tryptophan (W) at position 100 (NGF‐R100W). HSAN V patients experience a loss of pain and temperature sensation but with preserved cognitive functions, whereas HSAN IV patients with mutations in the *NTRK1* gene (encoding the TrkA receptor), on the other hand, exhibit both a loss of pain and temperature sensation as well as cognitive impairment. In line with this, in vitro studies comparing wild‐type NGF and the NGF‐R100E mutant demonstrate functional similarities (Covaceuszach et al., 2010).

To further investigate the NGF signalling mechanisms, we compare NGF with three NGF mutants and their abilities to induce phosphorylation of TrkA and the downstream signalling protein, the extracellular signal‐regulated protein kinases 1 and 2 (ERK1/2), along with proliferation and differentiation in several cell types. Increased understanding of NGF signalling is highly important for the development of a future neurotrophic therapy of Alzheimer's disease and other neurodegenerative disorders.

## 2 MATERIALS AND METHODS

### 2.1 Materials

Human foetal dorsal root ganglion (DRG) tissues originated from first trimester tissues (7–9 weeks postconception) collected with informed written consent after elective routine abortions. The procedure was approved by the Regional Ethical Committee, Stockholm (ethical approvals 2007/1477‐31/3, 2011/1101‐32 and 2013/564‐32), and the Swedish National Board of Health and Welfare. U2OS‐TrkA/p75‐SHC1 cells, U2OS‐TrkA‐PLCγ1 cells and PathHunter Detection Kit from DiscoverX and rat pheochromocytoma (PC12) cells from ATCC (LGC Promochem) were used. Human Phospho‐TrkA DuoSet IC ELISA and Phospho‐ERK1 (T202/Y204)/ERK2 (T185/Y187) DuoSet IC ELISA were from R&D Systems. Pierce^™^ Rapid Gold BCA Protein Assay Kit and Spectra Multicolor Broad Range Protein Ladder were from Thermo Fisher Scientific Inc. Black 384‐well poly‐D‐lysine (PDL)‐coated high‐content imaging plates with transparent bottom (cat. no 6007710) was from PerkinElmer and white assay 384‐well plates (#3570) from Corning. Anti‐β‐tubulin (G‐8) mouse monoclonal IgG3 antibody, 200 μg/ml, was from Santa Cruz Biotechnology, and phospho‐p44/42 MAPK (ERK1/2) (Thr202/Tyr204) rabbit monoclonal IgG1 antibody was from Cell Signaling Technology. Eagle's minimal essential medium (EMEM), collagen IV, formaldehyde solution, Hoechst 33258 solution and bovine serum albumin (BSA) were from Sigma‐Aldrich. Leibovitz's L15 medium, Dulbecco's modified Eagle's medium (DMEM), foetal bovine serum (FBS), horse serum, geneticin G418 and TrypLE Express were from Gibco Life Technologies. Penicillin‐streptomycin and Alexa Fluor 488 goat anti‐mouse IgG (H+L), 2 mg/ml, were from Invitrogen. NGF was purchased from PeproTech, whereas Acturum AB kindly provided NGF‐R100E, NGF‐W99A and NGF‐K95A/Q96A.

### 2.2 Expression and purification of NGF mutants

**Figure 1:**
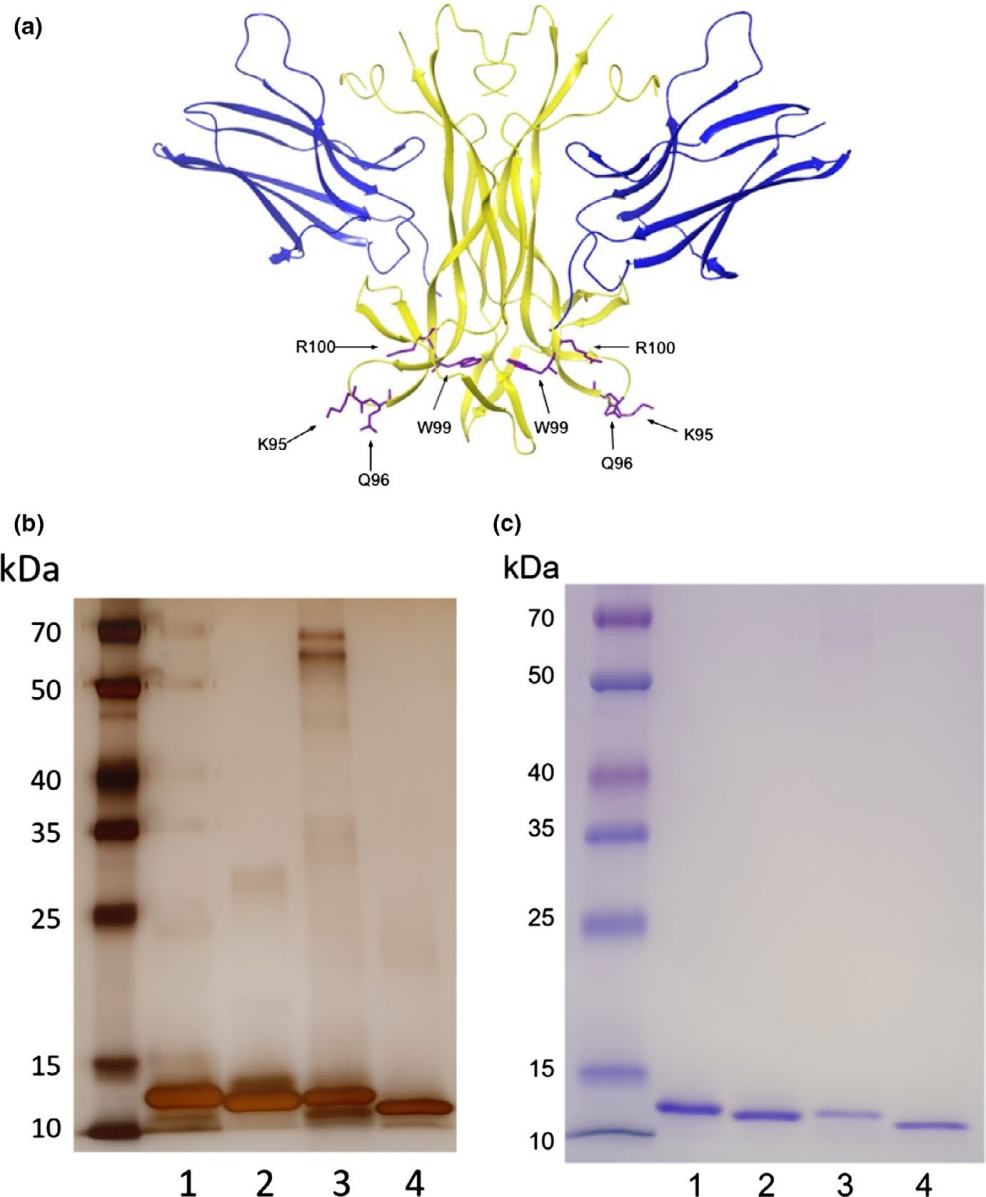
X‐ray structure of NGF/TrkA and SDS‐PAGE analysis of NGF mutants. (a) The interacting part of nerve growth factor (NGF) dimer (yellow) together with the extracellular D5 domain of the TrkA receptor (blue). The examined mutated residues (R100, W99 and K95/Q96) are numbered from the N‐terminus in the mature human NGF protein and are shown in purple. The coordinates and structure factors were from the Protein Data Bank, accession code 2IFG (Wehrman et al., 2007). (b) Silver staining and (c) Coomassie Blue staining of 250 ng wild‐type NGF or mutant NGF protein. From left to right, Spectra Multicolor Broad Range Protein Ladder, 1) wild‐type NGF, 2) NGF‐R100E, 3) NGF‐W99A and 4) NGF‐K95A/Q96A

The single NGF mutant NGF‐R100E (Capsoni et al., 2011; Covaceuszach et al., 2010) was chosen as a more stable variant of the HSAN V NGF mutant R100W. The NGF mutant NGF‐W99A was used to evaluate the effects of disruption of the lipid‐binding site on NGF. The double NGF mutant NGF‐K95A/Q96A was designed to study alterations of NGF loop 4. Figure 1a depicts the crystal structural of the NGF dimer in complex with the extracellular NGF‐binding domain of TrkA, where the amino acid residues of NGF studied in this report, K95/Q96, W99 and R100, are shown in purple.

Cloning, expression and purification of NGF constructs were performed at Sino Biological Inc. Briefly, human NGF mutants were cloned into *E. coli* expression plasmid pET30a‐pro‐EK. Verification of the authenticity of the subcloned cDNA was performed by restriction enzyme digest and sequencing. Recombinant plasmid DNA was used to transform BL21 (DE3) bacteria for expression. Approximately 10 L of the high expression clone was grown in enriched lysogeny broth (LB) medium and induced with isopropyl β‐D‐1‐thiogalactopyranoside (IPTG); the resulting cell pellet was harvested for protein purification. Briefly, bacteria were lysed by sonication and the resulting inclusion bodies were collected by centrifugation. Inclusion bodies were thereafter solubilized, and the proteins were refolded in refolding buffer (100 mM Tris‐HCl, pH 9.5, 240 mM NaCl, 10 mM KCl, 0.4 M sucrose, 0.75 M l‐arginine, 5 mM ethylenediaminetetraacetic acid (EDTA), 5 mM glutathione (GSH) and 0.5 mM glutathione disulphide (GSSG)) and thereafter dialysed (20 mM phosphate buffer, pH 7.0, 150 mM NaCl, 1 mM EDTA). Proteins were purified using a Sepharose High Performance column. Protein bound to the column was eluted in two steps, and more than 90% of refolded pro‐EK‐NGF protein was eluted in the second step. The eluate was desalted on a G‐25 column and then digested by addition of 6400 units of recombinant enterokinase (rEK). Analysis by sodium dodecyl sulphate–polyacrylamide gel electrophoresis (SDS‐PAGE) showed almost all of the pro‐EK‐NGF protein was digested, and the target NGF protein was clearly seen on the gel. A final purification step using a size‐exclusion column was performed, showing that the final product was >95% pure as judged by silver and Coomassie blue staining in SDS‐PAGE gels according to standard protocol (Figure 1b,c) and non‐aggregated (>90% as single peak at expected elution position on a size‐exclusion chromatography (SEC) column).

### 2.3 Human primary neuronal culture

DRG cells were isolated from human foetal ganglia originating from all spinal segmental levels. Dissections were performed under sterile conditions in 0.9% sodium chloride. The ganglia were collected in DMEM medium and disassociated within 1 hr. Media was removed and the DRG digested with TrypLE Express for 4 min at 37°C. Neurobasal medium supplemented with 2% B27 and 0.5 mM l‐glutamine was added, and the DRG was triturated with a plastic pipette tip. Media and TrypLE Express were removed, and the digestion process was repeated twice. Dissociated single cells were diluted in complete Neurobasal medium, stained with trypan blue and counted using a Bürker chamber and seeded in 384‐well PDL‐ and 0.01% fibronectin‐coated plates. Cells were plated at a density of 25,000 cells/ml (500 cells/well) in 20 μl complete Neurobasal medium. One hour after plating, 20 μl of media with serial dilution of wild‐type NGF or NGF mutants, ranging from 1 pg/ml to 100 ng/ml, was added and the plates were subsequently incubated at 37°C in humidified atmosphere with 5% CO_2_ for 4 days.

### 2.4 PC12 cell culture

PC12 cells were cultured in DMEM supplemented with 10% FBS, 5% horse serum and 1% penicillin‐streptomycin in a humidified incubator at 37°C and 5% CO_2_. Cells were maintained adherent in collagen IV‐coated flasks. For experimental procedures, PC12 cells were serum‐starved to evoke NGF‐dependent cell survival and differentiation using DMEM supplemented with 1% horse serum and 1% penicillin‐streptomycin. Cells were stained with trypan blue and counted using a Bürker chamber and seeded in 384‐well PDL/collagen IV‐coated plates at a density of 25,000 cells/ml (500 cells/well) in 20 μl assay media. One hour after plating, 20 μl of assay media with serial dilution of wild‐type NGF or NGF mutants was added, and the plates were subsequently incubated at 37°C in humidified atmosphere with 5% CO_2_.

### 2.5 Immunocytochemistry

Cells were fixed in assay media for 30 min at room temperature with 4% paraformaldehyde and then permeabilized with 0.3% Triton X‐100 in Tris‐buffered saline and blocked with 2.5% BSA in 0.3% Triton X‐100 in Tris‐buffered saline for 1 hr at room temperature. Cells were incubated overnight at 4°C with mouse anti‐β‐tubulin antibody (1:200 dilution in blocking solution) and for double staining with rabbit anti‐phospho‐p44/42 (pERK1/2) antibody (1:200 dilution in blocking solution). The immunolabelling was visualized with secondary antibodies conjugated to goat anti‐mouse Alexa‐488 (1:750) and goat anti‐rabbit Alexa‐647 (1:750). Cellular nuclei were examined with Hoechst nuclear stain, and cells were defined as β‐tubulin‐positive objects with nucleus and cell soma. Neurites were defined as β‐tubulin‐positive extensions from a valid cell body (with the presence of a cell nucleus surrounded by cytoplasm). To quantify the pERK1/2 average intensity, the total intensity in the Far‐red channel (Alexa‐647) was measured and the average intensity per cell was calculated. Images were collected on a Thermo Scientific Cellomics Array Scan V^TI^ HCS Reader, and high‐content imaging analysis was performed using Cellomics Scan Software. Cell number, average neurite length per cell in μm and pERK1/2 average intensity staining per cell were quantified. Cell proliferation was measured by quantifying the number of selected cells, and cell differentiation was assessed by quantifying neurite length per differentiated cell stained with anti‐β‐tubulin antibody. Results were exported as mean values per well.

### 2.6 U2OS‐TrkA chemiluminescence cell assay

The enzyme fragment complementation (EFC) technique used for measuring activation of the TrkA receptor has been described previously (Forsell et al., 2013). Briefly, the assay is a proximity‐based assay from DiscoverX where the interaction between the phosphorylated TrkA receptor at tyrosine 490 (Y490) and the intracellular signalling protein SHC1 (Src homology 2 domain containing transforming protein 1) is accompanied by complementation between two parts of β‐galactosidase, leading to an active β‐galactosidase enzyme. The activation of the TrkA receptor is quantified by measuring the amount of active β‐galactosidase which converts a non‐luminescent substrate into a luminescent product. There are several adaptor proteins known to mediate the effects of NGF via direct association with the intracellular domain of TrkA, for example SHC1, phosphoinositide 3‐kinase (PI3K) and phospholipase C gamma 1 (PLCγ1). With this in mind, we constructed an additional cell line that could be used to quantitate the interaction between phosphorylated TrkA at tyrosine 785 (Y785) and PLCγ1. Recombinant human osteosarcoma U2OS cells were maintained in EMEM supplemented with 10% FBS, penicillin/streptomycin and hygromycin/geneticin/puromycin. Cells were stained with trypan blue and counted using a Bürker chamber and then seeded in 384‐well plates in EMEM containing 0.5% horse serum. After seeding 20 μl cell suspension per well (350,000 cells/ml) in white tissue‐cultured treated plates (CulturePlate, PerkinElmer), plates were incubated for approximately 18 hr in humidified atmosphere with 5% CO_2_. The experiment was initiated by the addition of 10 μl of media with serial dilution of wild‐type NGF or NGF mutants to each well, followed by incubation for 3 hr at room temperature. After that, 5 μl of substrate was added and the plates were incubated for 60–90 min. Subsequently, total chemiluminescence was read at all wavelengths using an EnVision plate reader from PerkinElmer.

### 2.7 Human phospho‐TrkA and phospho‐ERK1/2 ELISA

Recombinant U2OS‐TrkA cells or PC12 cells were stained with trypan blue and counted using a Bürker chamber. The U2OS‐TrkA cells were seeded in EMEM containing 0.5% horse serum, at a concentration of 75,000 cells/well in 96‐well plates for analysis of phospho‐TrkA (pTrkA) or 100,000 cells/well in 96‐well plates for analysis of phospho‐ERK1/2 (pERK1/2). The PC12 cells were seeded in DMEM containing 0.5% horse serum, at a concentration of 333,000 cells/well in 24‐well plates for analysis of pERK1/2. 24 hours after plating, the cells were treated with serial dilution of NGF or mutant NGF, diluted in PBS containing 0.1% BSA, for 30 min in room temperature. Vehicle served as a control. The cells were lysed in 75 μl lysis buffer, and the phosphorylated TrkA or ERK1/2 levels were quantified using a solid‐phase sandwich enzyme‐linked immunosorbent assay (ELISA) according to the manufacturer's instruction (R&D Systems). A standard curve was generated for phospho‐TrkA or phospho‐ERK1/2, from which the total levels of each protein were quantified. Total protein levels in the samples were quantified using Pierce^™^ Rapid Gold BCA Protein Assay Kit according to the manufacturer's instruction (Thermo Fisher Scientific Inc.). Phospho‐TrkA or phospho‐ERK1/2 protein levels were normalized to 100 ng/ml wild‐type NGF.

### 2.8 Western blot analysis

DRG cells were seeded in 96‐well plates and maintained in culturing media for 4 days. Cells were washed once with phosphate‐buffered saline (PBS) and immediately exposed to 30 ng/ml wild‐type NGF, NGF mutant or vehicle (0.1% BSA in 1:1 PBS/Leibovitz's L‐15 medium) for 30 min in room temperature. Cells were washed once with PBS and subsequently suspended in radioimmunoprecipitation assay (RIPA) buffer supplemented with Complete protease inhibitor mix and phosphatase inhibitor mix. Cells were lysed for 10 min at 4°C, mixed with 4× NuPage sample buffer, sonicated and heated at 95°C. Proteins were loaded on Novex Bis‐Tris gel 4%–12% gel and run at 120 mV for 45 min using MES running buffer. The separated proteins were transferred from the gel to polyvinylidene difluoride (PVDF) membrane using an iBlot instrument (Invitrogen). After transfer, the membrane was blocked in Tris‐buffered saline (TBS)‐Tween + 5% milk powder for 60 min at room temperature. Primary antibody (phospho‐p44/42 MAPK (ERK1/2) (Thr202/Tyr204) rabbit monoclonal antibody) was diluted 1:2,000 in TBS‐Tween + 5% BSA and incubated with the membrane overnight at 4°C. The membrane was thereafter repetitively washed with TBS‐Tween. Secondary antibody (IRDye Rabbit 800CW, LI‐COR) was diluted 1:15,000 in 1:1 TBS‐Tween and LI‐COR blocking buffer and the membrane was incubated for 1 hr at room temperature, protected from light. The membrane was washed four times in TBS‐Tween, and near‐infrared (NIR) detection was performed on an Odyssey CLx Imaging System (LI‐COR Biosciences). After this, membranes were washed, treated with sodium azide, blocked and thereafter incubated with mouse monoclonal anti‐β‐actin antibodies and the analysis procedure was repeated once with secondary anti‐mouse antibodies.

### 2.9 Data analysis and statistical calculations

The software GraphPad Prism (GraphPad Software, Inc.) was used to calculate half‐maximal effective concentrations (EC_50_ values) using non‐linear regression analysis and Student's *t* test to compare EC_50_ values. One‐way ANOVA followed by *post hoc* Bonferroni multiple comparison test was used to compare responses represented in bar charts and for ELISA phospho‐TrkA levels, using wild‐type NGF as a comparison at each dose. To test whether ELISA phospho‐TrkA dose–response curves differ from each other, Prism non‐linear regression with extra sum‐of‐squares *F* test was used. Data are reported as mean values ± standard error of the mean (*SEM*), unless otherwise stated, and considered significant if *p* < .05.

## 3 RESULTS

### 3.1 Effects of wild‐type NGF and NGF mutants on proliferation and differentiation in human foetal dorsal root ganglion (DRG) neurons

**Figure 2:**
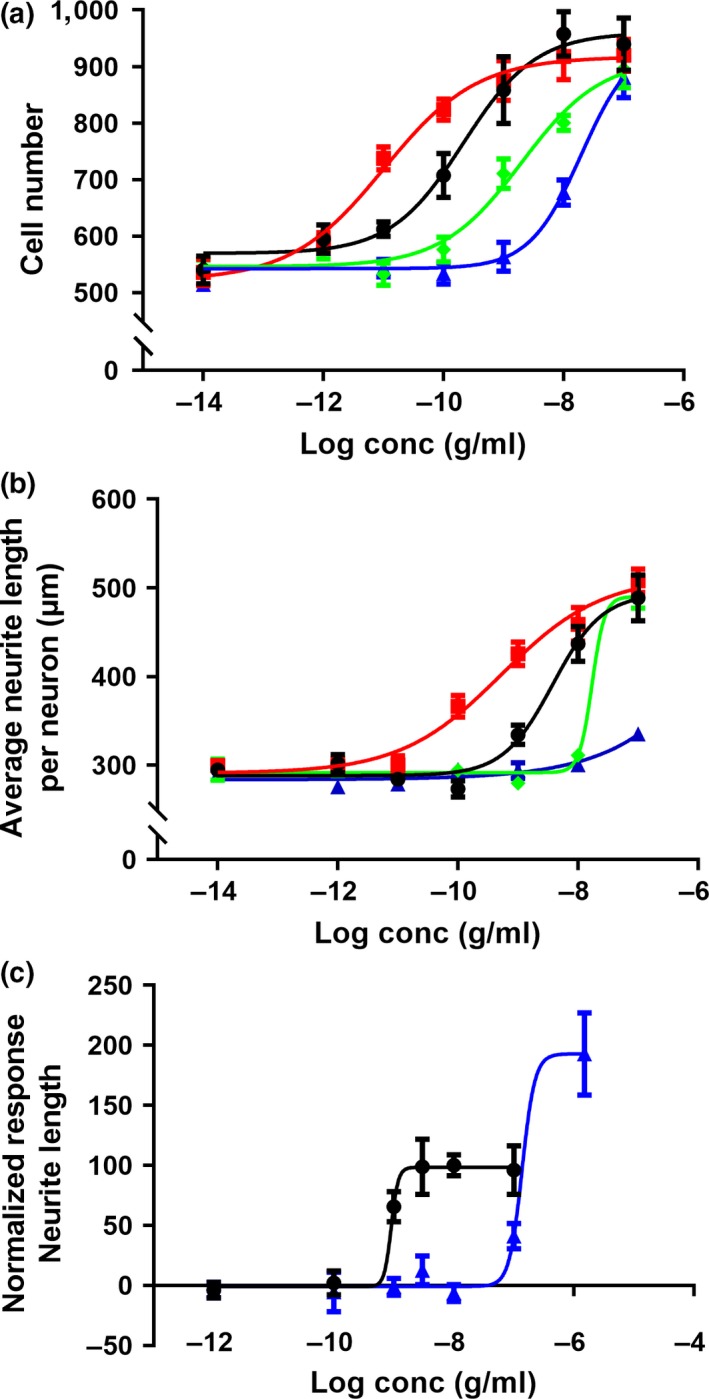
Dose–response stimulatory effects of wild‐type and mutant NGF on proliferation and differentiation in primary human foetal dorsal root ganglion (DRG) neurons. Immunofluorescent automatic high‐content image analysis was performed as described in Materials and Methods2. Human foetal DRG neurons were treated with 10‐fold serial dilutions of wild‐type NGF (black circles ●), NGF‐R100E (red squares 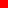), NGF‐W99A (green diamonds 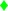) and NGF‐K95A/Q96A (blue triangles 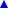) on day 0 and incubated for 4 days. Dose‐dependent effects of wild‐type or mutant NGF on (a) proliferation, as judged by Hoechst and β‐tubulin‐positive cells, or (b) differentiation, as judged by average neurite length per cell. (c) Normalized dose‐dependent effects of wild‐type NGF or NGF‐K95A/Q96A on differentiation at high concentrations. Three independent biological repeats with eight (a and b) or four (c) technical replicates were conducted

**Figure 3:**
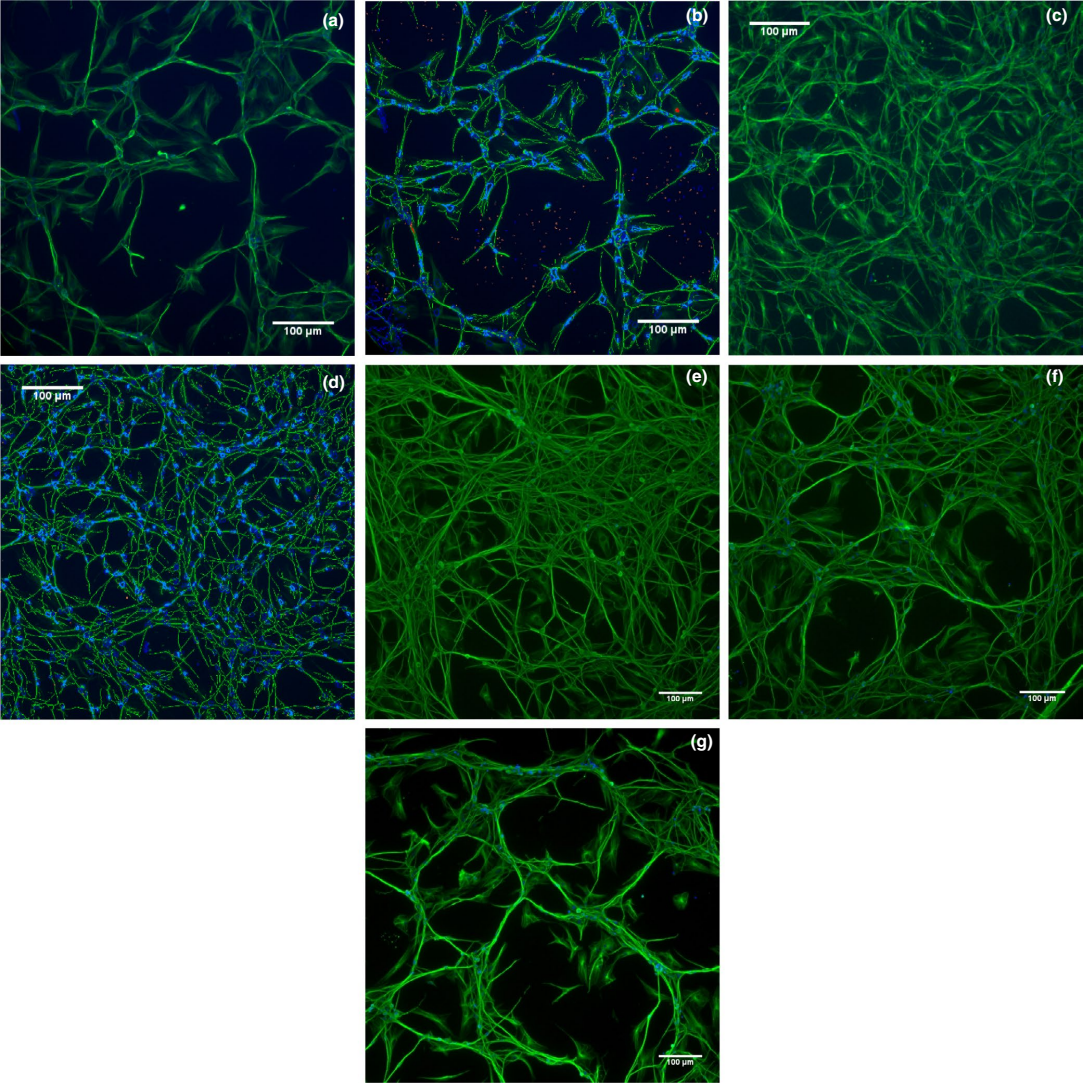
Immunocytochemistry images of human foetal dorsal root ganglion (DRG) neurons after 4 days in culture with NGF or NGF mutants. Human foetal DRG neuronal cells were grown in PDL‐ and fibronectin‐coated 384‐well plates for 4 days. Hoechst nuclear stain was used to label cell nuclei and mouse monoclonal IgG_3_ anti‐β‐tubulin (G‐8) antibody, and Alexa‐488 goat anti‐mouse IgG secondary antibody was used to label cell bodies and neurites. Immunofluorescent analysis of Hoechst (excitation wavelength 386 nm (blue)) and β‐tubulin (excitation wavelength 485 nm (green)) was performed using a Thermo Scientific Cellomics^®^ Array Scan VTI. Vehicle‐treated or 10 ng/ml wild‐type NGF‐treated DRG neuronal cell culture is displayed in (a) or (c), respectively, and analysis and quantification with the Neuronal Profiling BioApplication of the same image are shown in images (b) and (d). Dark blue colour in (b) or (d) indicates the presence of nuclei, light blue colour indicates the presence of β‐tubulin‐positive cell body, and green indicates the identified neurites. Rejected nuclei are shown in orange, and rejected cell bodies are shown in red. Images e–g show DRG neuronal cell culture treated with 10 ng/ml mutant NGF; R100E (e), W99A (f) and K95A/Q96A (g)

In order to investigate the effects of NGF or NGF mutants on proliferation and differentiation of human DRG, cells were incubated with increasing concentrations of the ligands as depicted in Figure 2. NGF‐R100E was the most potent of the three tested NGF mutants (Figure 2a,b), even more potent than wild‐type NGF, in terms of proliferation (*p* = .0495) and differentiation (*p* = .0339). The NGF‐W99A and NGF‐K95A/Q96A mutants, on the other hand, were less potent than wild‐type NGF to promote proliferation and differentiation. However, NGF‐W99A reached full activity at 30 ng/ml both regarding proliferation and differentiation, whereas NGF‐K95A/Q96A reached full proliferation activity at 30 ng/ml and exceeded the maximum level of differentiation compared with wild‐type NGF at 1.5 μg/ml (Figure 2c). The maximum neurite length per neuron of NGF‐K95A/Q96A was approximately twice that of wild‐type NGF in human foetal DRG neurons. Representative immunocytochemistry images using anti‐β‐tubulin antibody are shown in Figure 3 demonstrating the differentiation of human foetal DRG neurons treated with wild‐type NGF or NGF mutants at 10 ng/ml. NGF and the NGF mutants were more potent in stimulating proliferation as compared to differentiation (Table 1).

**Table 1:** Stimulatory effects of wild‐type NGF and NGF mutants on proliferation and differentiation in human foetal dorsal root ganglion (DRG) neurons

Human DRG neurons	Proliferation EC_50_ (ng/ml) Mean ± *SEM*	Differentiation EC_50_ (ng/ml) Mean ± *SEM*
Wild‐type NGF	0.69 ± 0.19	2.2 ± 0.61
NGF‐R100E	0.026 ± 0.017	0.19 ± 0.11
NGF‐W99A	5.9 ± 1.9	8.6 ± 3.7
NGF‐K95A/Q96A	15 ± 4.3	250 ± 110

Note

Proliferation and differentiation after 4 days in culture were measured and analysed using immunofluorescent automatic high‐content image analysis. EC_50_ values (ng/ml) are reported as the mean EC_50_ ± *SEM* from at least three independent biological repeats, each with at least four technical repeats.

### 3.2 Effects of wild‐type NGF and NGF mutants on proliferation and differentiation in PC12 cells

**Figure 4:**
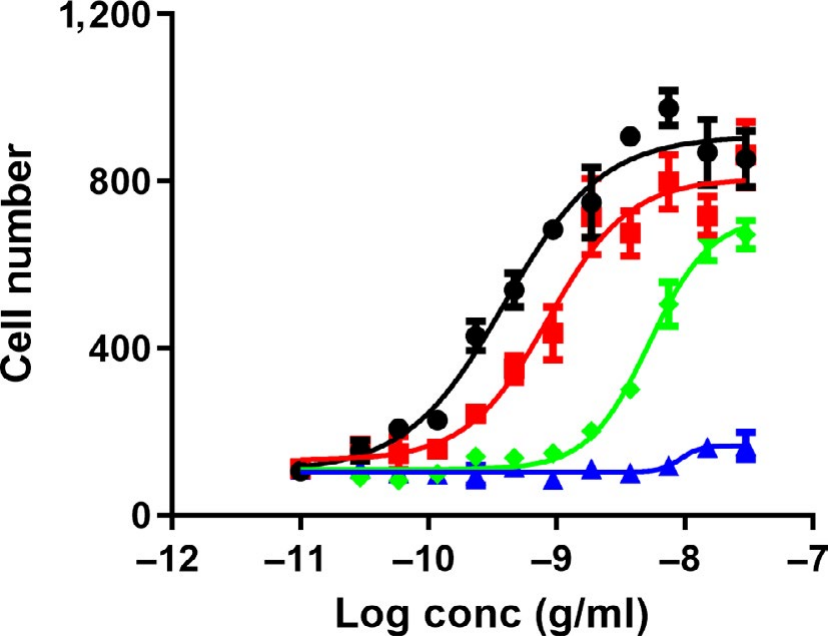
Effects of wild‐type NGF or NGF mutants on proliferation in PC12 cells. Immunofluorescent automatic high‐content image analysis was performed as described in Materials and Methods2. Data show dose‐related effects on number of cell bodies in PC12 cells after treatment with wild‐type NGF (black circles ●), NGF‐R100E (red squares 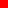), NGF‐W99A (green diamonds 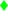) and NGF‐K95A/Q96A (blue triangles 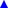) ranging from 30 pg/ml to 30 ng/ml after 4 days in culture. Seven independent biological repeats with four technical replicates were conducted

**Figure 5:**
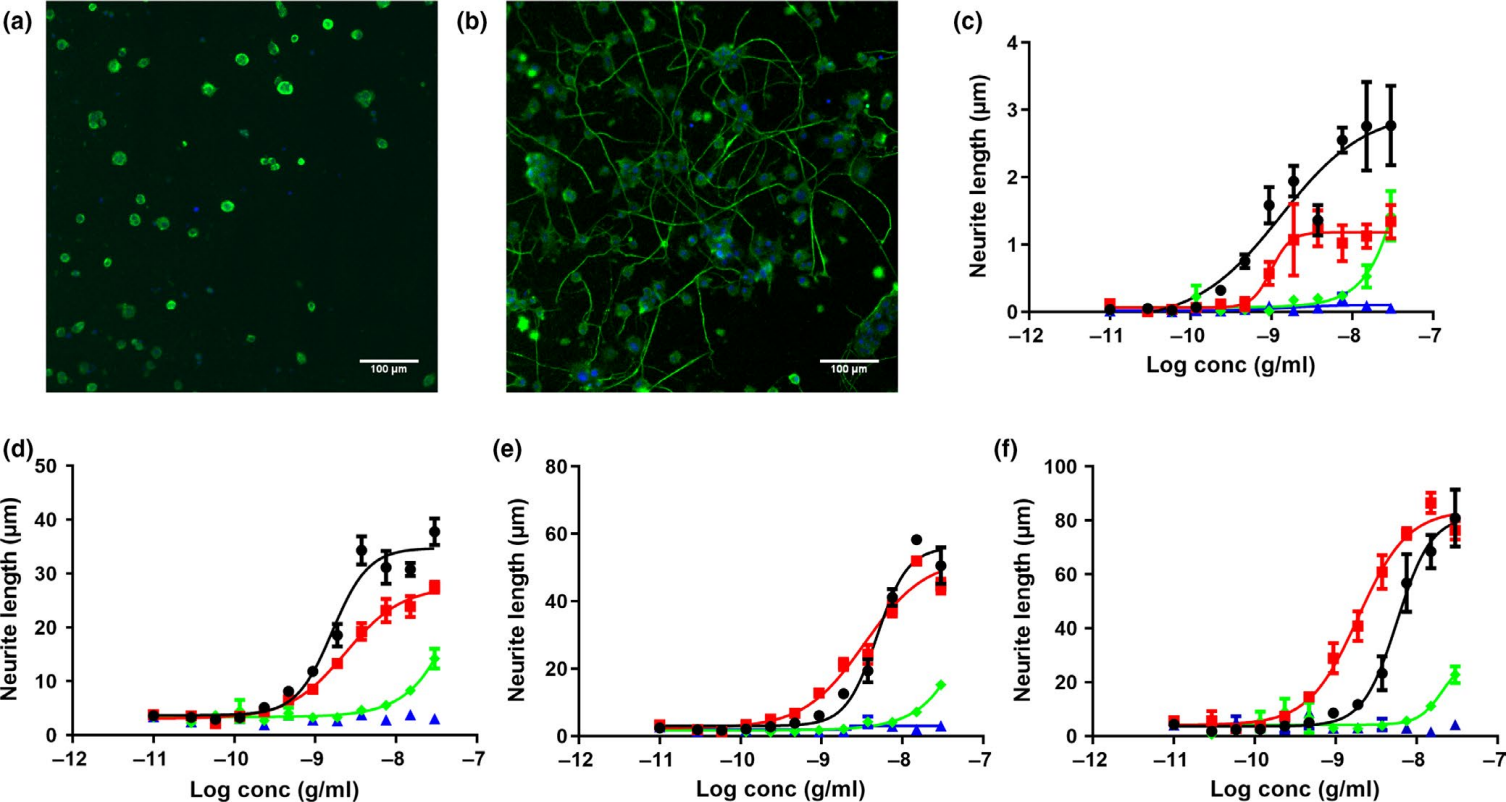
NGF‐induced differentiation in PC12 cells at different time points. Immunofluorescent automatic high‐content image analysis was performed as described in Materials and Methods2. Representative immunofluorescence images of PC12 cells stained with Hoechst (blue) and anti‐β‐tubulin antibody (green) in vehicle conditions (a) or 10 ng/ml wild‐type NGF treatment (b) for 4 days. (c–f) Dose‐related effects on differentiation (neurite length per cell in μm) at different time points (2–8 days of incubation) in PC12 cells in the presence of wild‐type NGF (black circles ●), NGF‐R100E (red squares 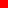), NGF‐W99A (green diamonds 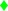) and NGF‐K95A/Q96A (blue triangles 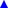) ranging from 30 pg/ml to 30 ng/ml. Two independent biological repeats with four technical replicates were conducted

Wild‐type NGF and NGF‐R100E mutant displayed similar proliferative effects in NGF‐dependent serum‐starved PC12 cells. NGF‐W99A was approximately 15 times less potent to promote proliferation, and NGF‐K95A/Q96A only reached 10% efficacy regarding potentiation of proliferation of what could be observed for wild‐type NGF (Figure 4, Table 2). The effects of NGF or mutant NGF on PC12 cell differentiation were determined after 2–8 days of incubation (Figure 5). Wild‐type NGF and NGF‐R100E induced neurite outgrowth in a dose‐dependent manner. NGF‐R100E had a slower onset to promote neurite outgrowth and was less effective as compared to wild‐type NGF up to 4 days in culture, but prolonged incubation time up to 8 days demonstrated that NGF‐R100E was more potent to promote differentiation than wild‐type NGF (Figure 5). The NGF‐W99A mutant displayed a small but a positive effect on neurite length at the highest doses tested. The double mutant NGF‐K95A/Q96A, however, had no ability to induce neurite outgrowth in PC12 cells. As observed for DRG neurons, wild‐type NGF and the NGF mutants were more potent with respect to increase proliferation than to stimulate differentiation in PC12 cells (Table 2).

**Table 2:** Stimulatory effects of wild‐type NGF and NGF mutants on proliferation and differentiation in PC12 cells

PC12 cells	Proliferation EC_50_ (ng/ml) Mean ± *SEM*	Differentiation EC_50_ (ng/ml) Mean ± *SEM* Mean ± *SEM*
Wild‐type NGF	0.17 ± 0.051	3.3 ± 0.42
NGF‐R100E	0.32 ± 0.14	3.1 ± 0.25
NGF‐W99A	2.7 ± 0.71	NA
NGF‐K95A/Q96A	NA	NA

Note

Proliferation and differentiation after 4 days in culture were measured and analysed using immunofluorescent automatic high‐content image analysis. EC_50_ values (ng/ml) are reported as the mean EC_50_ ± *SEM* from at least seven independent biological repeats, each with four technical repeats. Not applicable (NA) indicated EC_50_ values are of very poor potency and/or efficacy.

### 3.3 Activation of TrkA

**Figure 6:**
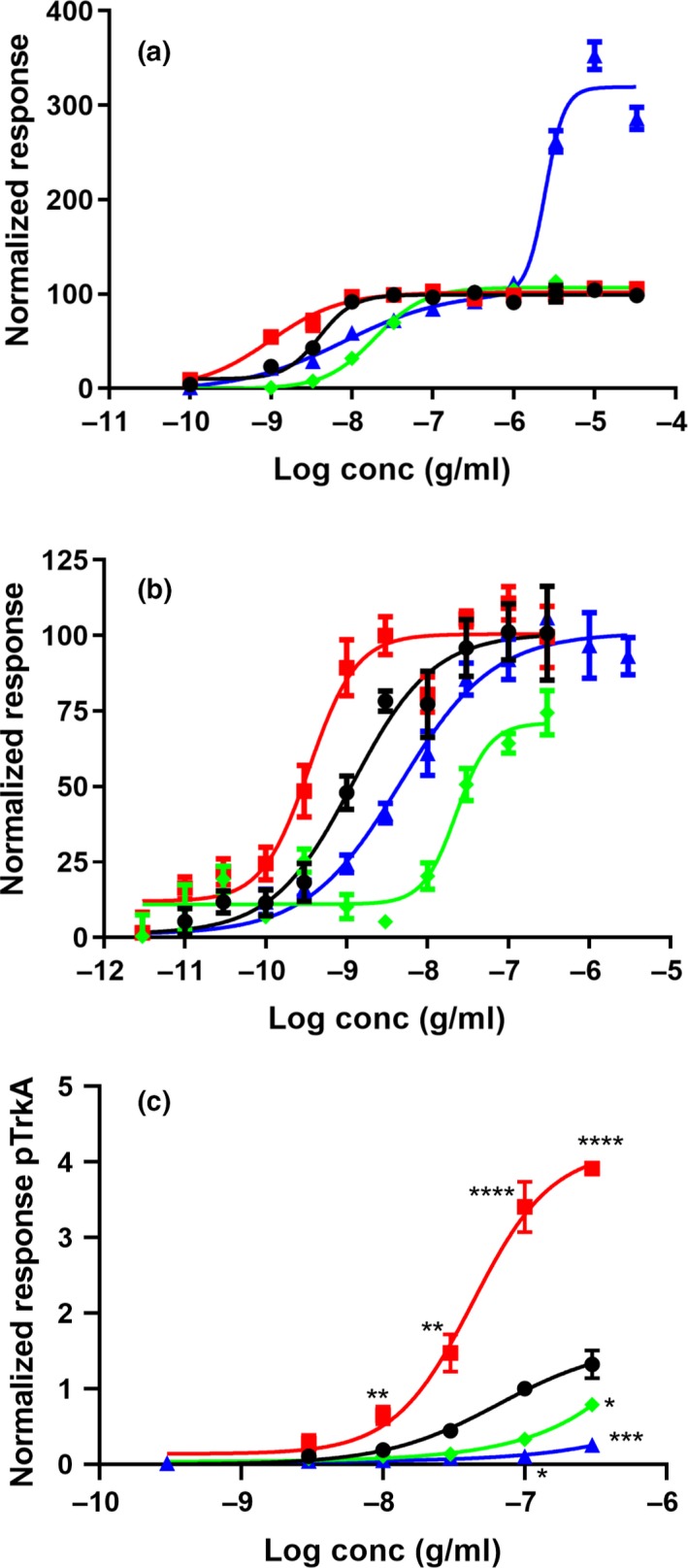
Activation of TrkA in U2OS‐TrkA/p75‐SHC1 cells. Total chemiluminescent signal from all wavelengths was measured using reagents and recombinant cells from DiscoverX. The luminescent signal is proportional to the amount of interactions between TrkA and SHC1 or PLCγ1 present in each well. Normalized response of the interaction between TrkA and SHC1 in U2OS‐TrkA/p75‐SHC1 cells (a), or between TrkA and PLCγ1 in U2OS‐TrkA‐PLCγ1 cells (b) after 3 hr of treatment. Five (a) or four (b) independent biological repeats with four technical replicates were conducted. (c) ELISA was used to quantify direct phosphorylation of TrkA from three independent biological repeats after normalization to 100 ng/ml wild‐type NGF. Results are mean ± *SEM*, significant alterations compared with wild‐type NGF were found for 100 ng/ml and 300 ng/ml R100E (****p < .0001), 30 ng/ml R100E (**p = .0016), 10 ng/ml R100E (**p = .0031), 300 ng/ml W99A (*p = .0363), 100 ng/ml K95A/Q96A (*p = .0185) and 300 ng/ml K95A/Q96A (***p = .0006). Wild‐type NGF (black circles ●), NGF‐R100E (red squares 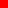), NGF‐W99A (green diamonds 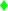) or NGF‐K95A/Q96A (blue triangles 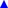)

Activation of TrkA at tyrosine 490 (Y490) and the subsequent recruitment of the SHC1 adaptor protein were measured as described previously (Forsell et al., 2013) using the U2OS‐TrkA/p75‐SHC1 chemiluminescent cell assay from DiscoverX. NGF‐R100E was significantly more potent to enhance activation of TrkA and to induce SHC1 recruitment (*p* = .0395) than wild‐type NGF (Figure 6a, Table 3). NGF‐W99A and NGF‐K95A/Q96A were less potent than wild‐type NGF to activate TrkA (Figure 6a, Table 3). The double mutant NGF‐K95A/Q96A displayed a second activation phase of the TrkA receptor. This second phase plateaued at approximately 300% of the activity of wild‐type NGF at around 10 μg/ml NGF‐K95A/Q96A (Figure 6a), and the second phase EC_50_ value is approximately 3 μg/ml. This second activation phase was not observed when using wild‐type NGF, NGF‐W99A or NGF‐R100E. The effects of wild‐type NGF and NGF mutants on U2OS‐TrkA‐PLCγ1 cells were analogous to the results obtained with U2OS‐TrkA/p75‐SHC1 cells, with same order of potency but without the second activation phase for NGF‐K95A/Q96A (Figure 6b).

**Table 3:** Stimulatory effects of wild‐type NGF and NGF mutants on activation of TrkA and the subsequent recruitment of the SHC1 protein in recombinant U2OS‐TrkA/p75‐SHC1 cell line

U2OS‐TrkA/SHC1 cells	Interaction between TrkA and SHC1 EC_50_ (ng/ml) Mean ± *SEM*
Wild‐type NGF	4.2 ± 1.0
NGF‐R100E	0.56 ± 0.21
NGF‐W99A	23 ± 7.5
NGF‐K95A/Q96A	20 ± 7.7

Note

Activation of TrkA was analysed by measuring the phosphorylation of TrkA at position Y490 and the concomitant docking of SHC‐1 to TrkA‐pY490 after 3 hr of treatment with wild‐type NGF or NGF mutants in the U2OS‐TrkA/p75‐SHC1 cells using the DiscoverX PathHunter assay. EC_50_ values (ng/ml) are reported as the mean EC_50_ ± *SEM* from five independent biological repeats, each with four technical repeats.

Phosphorylation of TrkA was verified using a phospho‐TrkA ELISA (Figure 6c). NGF‐R100E could induce phosphorylation of TrkA with a significantly higher efficacy than wild‐type NGF at all concentrations from 10 to 300 ng/ml. Non‐linear regression analysis with extra sum‐of‐squares F test confirmed that the dose–response curve for NGF‐R100E was significantly (*p* < .0001) separated from wild‐type NGF. The dose–response curves of NGF‐W99A and NGF‐K95A/Q96A using phospho‐TrkA ELISA did not plateau and curve comparisons could not be performed, but NGF‐K95A/Q96A displayed a significant change of TrkA phosphorylation at doses of 100 and 300 ng/ml and NGF‐W99A displayed a significant change at 300 ng/ml as compared to wild‐type NGF.

### 3.4 NGF‐dependent phosphorylation of ERK1/2

**Figure 7:**
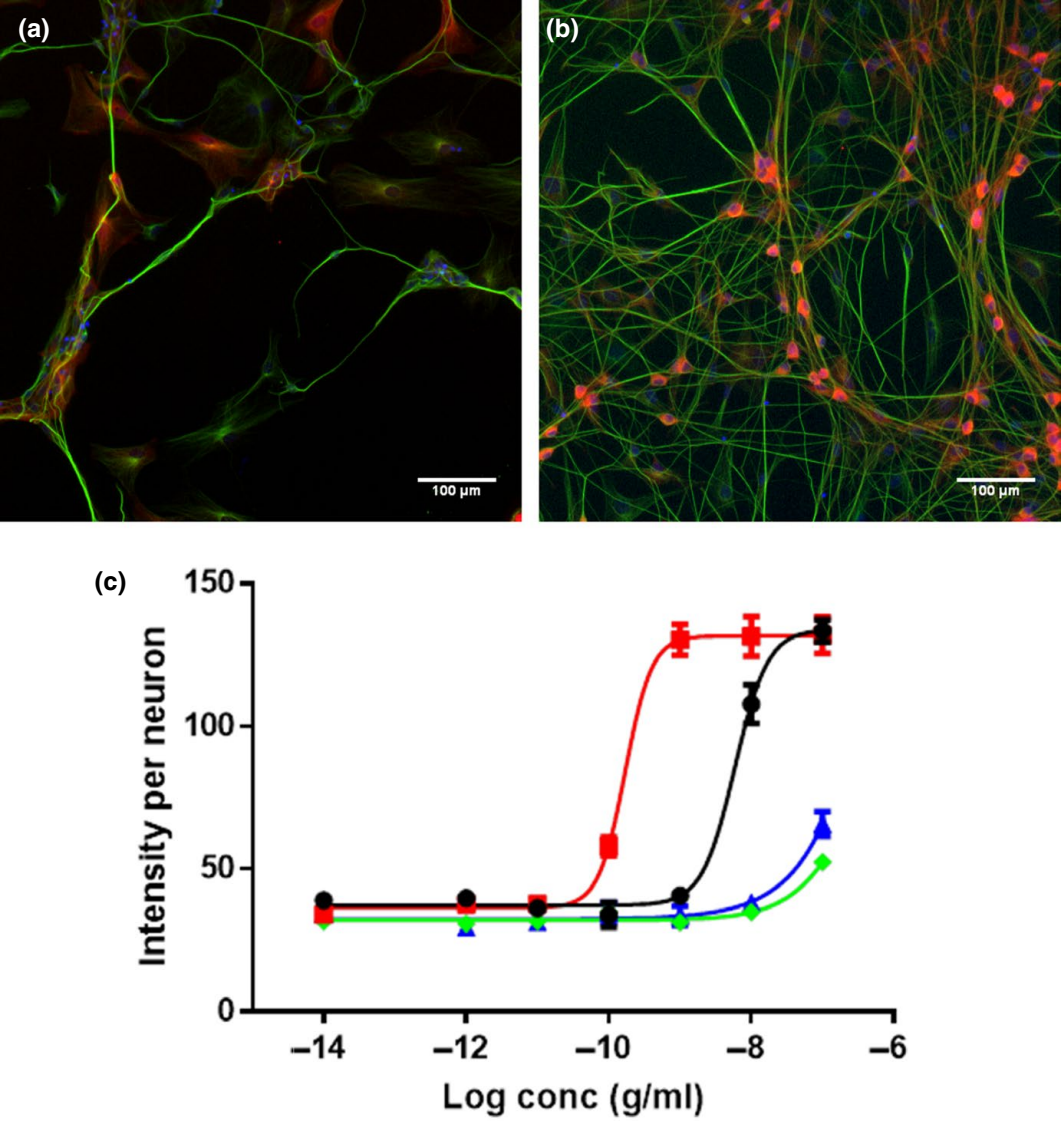
Immunocytochemical analysis of ERK1/2‐phosphorylation in human foetal dorsal root ganglion (DRG) neurons. Immunofluorescent automatic high‐content image acquisition was performed using an Array Scan VTI (Thermo Scientific Cellomics^®^), and quantification of nuclei, cell bodies and pERK1/2 staining was performed using the Neuronal Profiling V4 BioApplication Software. Representative immunofluorescence images of human foetal DRG neurons in vehicle‐treated cultures (a) or 100 ng/ml wild‐type NGF‐treated cultures (b) were stained with Hoechst nuclear stain to label cell nuclei (blue), anti‐β‐tubulin antibody to label cell bodies and neurites (green) and anti‐phospho‐p44/42 MAPK T202/Y204 antibody to stain pERK1/2 (red). (c) Average intensity per neuron of pERK1/2 staining in human DRG neurons after 4 days of incubation with wild‐type NGF (black circles ●), NGF‐R100E (red squares 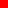), NGF‐W99A (green diamonds 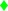) and NGF‐K95A/Q96A (blue triangles 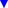). EC_50_ values for wild‐type NGF and NGF‐R100E are significantly different (Student's *t* test), *p* < .0001, while EC_50_ values for NGF‐W99A and NGF‐K95A/Q96A are not applicable as the phosphorylation of ERK1/2 was very low compared with wild‐type NGF. Three independent biological repeats with eight technical replicates were conducted

**Figure 8:**
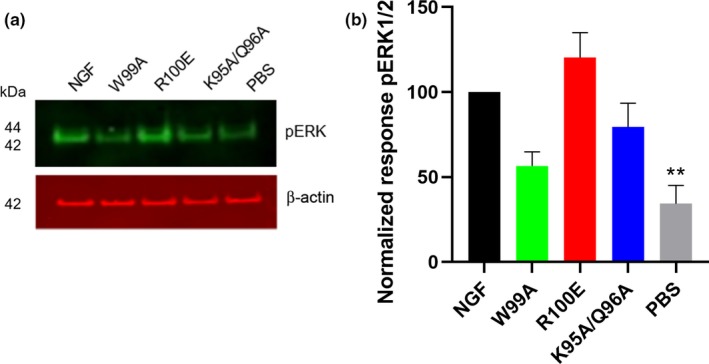
Western blot analysis of ERK1/2‐phosphorylation in human foetal dorsal root ganglion (DRG) neurons. (a) One representative Western blot analysis of three different experiments is shown. The DRG neurons were treated with 30 ng/ml wild‐type NGF, NGF mutants or PBS (vehicle) for 30 min. (b) Phospho‐ERK levels in human foetal DRG neurons were quantified using the anti‐pERK1/2 antibody (anti‐phospho‐p44/42 MAPK T202/Y204 antibody), and the results were normalized to the levels of β‐actin and wild‐type NGF. PBS (vehicle)‐treated DRG neurons displayed significantly lower levels of pERK1/2 (**p = .0066) compared with wild‐type NGF. The results are presented as mean ± *SEM* of three independent biological repeats

**Figure 9:**
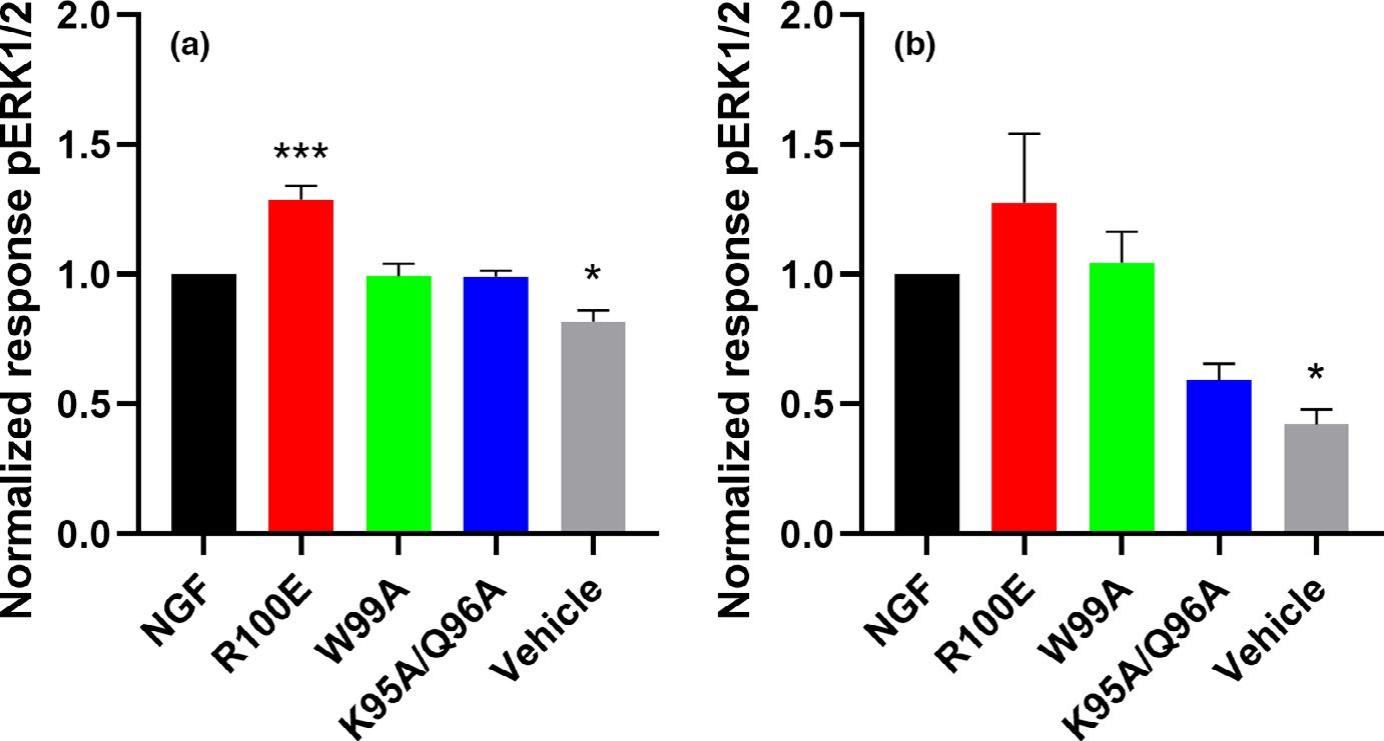
Phospho‐ERK1/2 levels in cultured U2OS‐TrkA/p75‐SHC1 cells and PC12 cells investigated with ELISA. Analysis of pERK1/2 levels in U2OS‐TrkA cells (a) or PC12 cells (b) after NGF stimulation was performed with ELISA. There were significantly increased pERK1/2 levels for NGF‐R100E (***p = .0005) compared with wild‐type NGF and significantly lower levels for vehicle‐treated U2OS‐TrkA/p75‐SHC1 cells (*p = .0121, a). In b, significantly lower pERK1/2 levels for vehicle‐treated PC12 cells compared with wild‐type NGF were found (*p = .0121). The results are presented as mean ± *SEM* from four independent biological repeats with one to twelve technical replicates

Immunocytochemical analysis of pERK1/2 levels in human foetal DRG neurons demonstrates that NGF‐R100E is significantly more potent than wild‐type NGF to induce phosphorylation of ERK1/2 in human foetal DRG neurons (*p* < .0001), when comparing EC_50_ values from three independent biological repeats (Figure 7). NGF‐W99A and NGF‐K95A/Q96A could only potentiate phosphorylation of ERK1/2 to a low extent at the highest dose tested (100 ng/ml). Western blot analysis of human foetal DRG neurons (Figure 8) and ELISA analysis of U2OS‐TrkA/p75‐SHC1 cells and PC12 cells (Figure 9) showed that NGF‐R100E was the most effective of the mutants and significantly more effective than wild‐type NGF regarding phosphorylation of ERK1/2 in U2OS‐TrkA/p75‐SHC1 cells (***p = .0005, Figure 9a).

## 4 DISCUSSION

An increasing number of studies support the notion that NGF and TrkA are promising targets for new therapeutics for the treatment of Alzheimer's disease (Allen, Watson, & Dawbarn, 2011; Cattaneo & Calissano, 2012; Iulita & Cuello, 2015; Xu, Wang, & Jin, 2016). However, due to the pleiotropic effects of NGF there are concerns for side effects when developing molecules that intervene with the NGF/TrkA pathway. In order to gain more insight into NGF‐dependent TrkA signalling, we have in this study compared different modalities to study the effects of NGF mutants, using human DRG neurons, PC12 cells and recombinant U2OS cells overexpressing TrkA. Human DRG neurons, which are dependent on NGF for survival and differentiation, are more likely to represent a relevant model of the functional effects of human neuronal cells in vivo than transformed cell lines or cells overexpressing the recombinant receptor. Moreover, as rat TrkA and human TrkA only are 86% identical in their primary amino acid sequence, with the majority of differences being found in the extracellular domain, the use of human TrkA or human cells expressing endogenous TrkA is important for studies of biological responses of human NGF and human NGF mutants. While recombinant cell lines provide an opportunity to directly study the interaction between phosphorylated TrkA and the recruitment of the first adaptor proteins in the signalling cascade, the signalling cascade downstream of TrkA can, however, differ markedly between cells and conditions, so for functional aspects, primary human foetal DRG neurons and possibly PC12 cells are more suitable than recombinant cells.

We show that the NGF‐R100E mutant displays significantly more potent stimulating effects on proliferation, differentiation and ERK1/2 phosphorylation in human foetal DRG neurons compared with wild‐type NGF, and in addition, NGF‐R100E is also significantly more potent in activating TrkA in U2OS‐TrkA cells. NGF‐W99A displayed a lower potency than wild‐type NGF in promoting proliferation, differentiation and phosphorylation of TrkA or ERK1/2. The NGF‐K95A/Q96A mutant exhibited diverging properties, with overall lower potency compared with wild‐type NGF to activate TrkA and to promote functional properties but with increased efficacy to generate neurite outgrowth in human foetal DRG neurons and also increased ability to enhance phosphorylation of TrkA at the SHC1‐site in U2OS‐TrkA‐SHC1 cells at elevated concentrations, compared with wild‐type NGF.

Biological activity of NGF mutants at positions K95, Q96, W99 and R100 has been investigated by others (Guo, Meyer, Kaur, Gao, & Neet, 1996; Ibáñez, Hallböök, Ebendal, & Persson, 1990; Ibáñez et al., 1992), but special interest was evoked when the R100W mutant in HSAN V was described in 2004 (Minde et al., 2004). In these patients, the pain‐mediated effect of NGF is reduced whereas the neurotrophic support function is intact (Covaceuszach et al., 2010; Minde et al., 2004). In addition to mutant NGF, other molecules, such as lipids or small dimeric peptides, may also generate differential signalling of NGF via TrkA (Gudasheva et al., 2015; Tong et al., 2012). Loop 4 of NGF (amino acid residue 91–97), which is known to be of importance for neurite outgrowth, is in close proximity to the amino acid residues W99 and R100, suggesting that this part of NGF is important in mediating effects on proliferation and differentiation. By mutating amino acids within this short sequence of the NGF protein, we could study the influence of specific amino acids on the functional responses of NGF.

The activation of TrkA by wild‐type NGF or the NGF mutants was comparable in the two U2OS‐TrkA cell lines, suggesting that the phosphorylation of Y490 and Y785 on TrkA is affected equally by the different mutants, except for the second activation phase of NGF‐K95A/Q96A present in U2OS‐TrkA/p75‐SHC1 cells. The fact that all the studied NGF mutants could induce phosphorylation of Y490 and Y785, but demonstrated differential effects on proliferation and differentiation, suggests that additional mechanisms are involved in regulating this response.

Human foetal DRG neuron immunocytochemistry images, shown in Figure 3, demonstrate that 4 days after seeding, distinct clusters of cells are formed within the wells treated with NGF‐K95A/Q96A or vehicle, while cells treated with wild‐type NGF or NGF‐R100E remain evenly distributed. We interpret this migration and clustering of cells as a sign of a survival reaction, whereby compromised neurons can increase survival by forming groups of cells that benefit from trophic support by the surrounding cells and engage in coordinated neuronal firing. An in vivo situation that could support this idea is the Hebbian theory, where repeated activities are thought to induce long‐lasting changes within the brain, and where cells in close proximity can fire together. As a consequence, axonal growth from such a group of neurons may be improved enough to reach other clusters of cells, despite the lack of neurotrophic support in terms of NGF found in these wells (Hebb, 1949; Munno & Syed, 2003). This phenomenon was not detected in PC12 cells. The temporal effects of NGF‐R100E seen in PC12 cells also indicate that caution should be taken when comparing TrkA signalling events, as the length of incubation can have pronounced effects on the response. Another aspect drawn from this study is the low capacity of the NGF‐W99A mutant to induce cell differentiation, but not proliferation, in PC12 cells compared with primary human foetal DRG neurons, which highlights the importance of the choice of in vitro cell culture system. As illustrated in our study, initiation of differentiation in PC12 cells may be hindered by their non‐neuronal nature and display a divergent result compared with human foetal DRG neurons.

It has previously been reported that NGF‐R100E is less effective than NGF to activate the TrkA/PLCγ1/ERK1/2 pathway (Capsoni et al., 2011). Still, other studies have shown that decreased signalling of the NGF‐R100W is due to low secretion of the mature mutant protein in certain cell types (Larsson, Kuma, Norberg, Minde, & Holmberg, 2009), rather than suppressed binding to the TrkA receptor. Our results imply that exogenously added NGF‐R100E has full, and even increased, capacity compared with wild‐type NGF to activate the TrkA receptor in human cultured cells.

Our results using the NGF‐W99A mutant demonstrate that tryptophan 99 is important for promoting neurite outgrowth in both human DRG neurons and PC12 cells. In addition, the two NGF residues K95 and Q96 are also essential for neurite outgrowth as revealed by the low potency or lack of neurite outgrowth when using the double mutant NGF‐K95A/Q96A in human DRG neurons or PC12 cells. It is also noteworthy that both NGF‐W99A and NGF‐K95A/Q96A have reduced capacity to induce phosphorylation of ERK1/2 in human foetal DRG neurons. This reduced ability to mediate phosphorylation of ERK1/2 could be an explanation for their lower potency to induce neurite outgrowth. It has previously been demonstrated that MAPK/ERK signalling pathway is essential for NGF‐induced differentiation, but not for NGF‐induced proliferation of PC‐12 cells (Klesse, Meyers, Marshall, & Parada, 1999; Pang, Sawada, Decker, & Saltiel, 1995).

In conclusion, we have demonstrated that NGF‐R100E has improved functional properties as compared to wild‐type NGF in human foetal DRG neurons, which is supported by phospho‐TrkA and phospho‐ERK1/2 activation in U2OS‐TrkA cells, PC12 cells and human foetal DRG neurons. We have identified different sites on NGF that appear to have a critical function for the NGF‐induced TrkA signalling, leading to altered phosphorylation of TrkA and ERK1/2 and the induction of neurite outgrowth by NGF or NGF mutants in human DRG neurons. Our study emphasizes the importance of studying functional responses in relevant in vitro cell models when evaluating neurotrophins and their effect on specific signalling pathways. These findings promote future research on the NGF‐R100E mutant in the treatment of Alzheimer's disease and other neurodegenerative disorders.

## CONFLICT OF INTEREST

G.N. and P.F. are employed by AlzeCure Pharma AB.

## AUTHOR CONTRIBUTIONS

M.D., G.N., M.E. and P.F. designed the study. M.D., E.S., E.Å., G.T. and P.F. performed the laboratory work; M.D., G.N. and P.F. analysed data; and M.D., G.N., E.S., M.E. and P.F. drafted the manuscript. All authors reviewed and revised the article for important intellectual content. All authors read and approved the final manuscript.

## Data Availability

The data are available on request by the authors.
