# Supplementary material for: Identification of amino acid residues of nerve growth factor important for neurite outgrowth in human dorsal root ganglion neurons
Source: Eur J Neurosci. 2019 Aug 1;50(9):3487–501. doi: 10.1111/ejn.14513 (PMC6899756; doi:10.1111/ejn.14513)
Supplement: Supplementary file 1 [file EJN-50-3487-s001.pdf]

EJN European Journal of Neuroscience FENS Federation of European Neuroscience Societies

Identification of amino acid residues of nerve growth factor important for neurite outgrowth in human dorsal root ganglion neurons

Forsell, Pontus (contact); Dahlström, Märta; Nordvall, Gunnar; Sundström, Erik; Åkesson, Elisabet; Tegerstedt, Gunilla; Eriksdotter, Maria

---

Review timeline:

Submission date: 23-Aug-2018
Editorial Decision: Reject – resubmission invited
Revision Received: 21-Dec-2018
Editorial Decision: Major Revision
Revision Received: 27-Jun-2019
Accepted: 05-Jul-2019

Editor: Paul Bolam
Reviewer 1: Jessica Kwok
Reviewer 2: Chengbiao Wu

1st Editorial Decision 26-Oct-2018

Dear Dr. Forsell,

First of all, apologies for the time it has taken to deal with your manuscript. We had difficulty engaging reviewers but have decide to go ahead with the comments of a single reviewer together with our thoughts on reading the manuscript. Based on this, we regret to inform you that we are not able to accept your manuscript for publication in EJN in its present form. However, the research described in your manuscript is potentially of interest and therefore we invite you to resubmit a much revised version.

As you can see below, the reviewer makes a series of comments that we are essentially in agreement with. These relate to the description of both the methods and the presentation of the results. Please carefully address each of the points that he/she has made and make revisions accordingly.

We note the following points that will need to be addressed in the re-submitted version.
- There are too many abbreviations in abstract for it to be accessible to the general readership of EJN
- The figures need to be of a higher resolution for publication.
- In accordance with EJN policy, please replace bar charts with more informative scatter plots or similar.
- Please include a graphical abstract with text
- Ensure that the reporting of statistical data adheres to EJN guidelines, notably report precise P valuses.
- Include direction and magnitude of changes/differences in the text as well as the statistical data.
- Typo '...incubated with different amountS of wild-type NGF or NGF...'
- When 'cell number' is referred to in the figures indicate what this means. There is no description of cell counting in the methods except to say that it was performed. Similarly, as pointed out by the reviewer, much more detail about measurement of neurite lengths.
- It is not transparent as to how many experiments/replications were performed. For example in Fig 4: 'Each point represents mean ± SEM (n = 4) from one representative experiment.'; it is not clear what this means, why only a representative example is reported or how we know it is representative. Figure 6 is even more confusing 'Each point represents mean ± SEM (n = 4) from one representative experiment from at least four independent experiments'.

- In general, the figure legends need to better describe the figures.

When revising the manuscript for re-submission, please embolden or underline major changes to the text so they are easily identifiable and please don't leave 'track change' formatting marks in your paper. If the changes made are extensive, please also provide an unmarked version.

Please provide a detailed response to the reviewers' comments, describing the changes you have made in the text. Please upload this response letter as a separate Word (.doc) file using the file designation "Authors' Response to Reviewers" when uploading your manuscript files.

When finalized, please upload your complete revised manuscript onto the website as a Word (.doc or .docx), or .rtf file. Please also ensure that a complete set of tables and figures is included as separate files, even if these have not changed from the originals. At this stage it is necessary to provide high resolution figures. Please see important instructions below.

To re-submit your manuscript, please go to your Author Centre and check in 'Manuscripts with decisions' under the previous manuscript number for a 'Create a resubmission' link. Also, please note that only the Author who submitted the original version of the manuscript should submit a revised version.

If you feel that your paper could benefit from English language polishing, you may wish to consider having your paper professionally edited for English language by a service such as Wiley's at http://wileyeditingservices.com. Please note that while this service will greatly improve the readability of your paper, it does not guarantee acceptance of your paper by the journal.

Thank you for submitting your work to EJN.

Best wishes,

Paul Bolam & John Foxe
co-Editors in Chief, EJN

Reviews:

Reviewer: 1

Comments to the Author
Dear editor,

The manuscript prepared by Dahlstrom et al. on "Identification of amino acid residues of nerve growth factor important for neurite outgrowth in human dorsal root ganglion neurons" attempted to interrogate the amino acid sequence of nerve growth factor (NGF) which are responsible for its physiological function, namely to support and maintain the survival/growth of neurons.

The authors have created three NGF mutants and assess their ability to induce the growth of DRG neurons and PC12 cells. Downstream activation of TrkA and Erk monitored. The authors reported that both NGF-R100E and NGF-K95A/Q96A mutations gave the strongest growth promoting response. The topic is exciting

and could potentially lead to very interesting findings.

However, there are places where experimental procedures or findings were not clearly explained and thus made it difficult to conclude the results. Most of the findings were reported in graphs (Fig. 2,4,5,6,7), and expect figure 2 where the cell images were then shown in figure 3, most of the other figures did not have accompanying images to support the quantification. This is particularly important when intensity measurement was performed on the neurons, such as fig 7.

In Figure 2 and 3 where DRG were used for the experiments, the authors claimed that the NGF (wildtype and some mutant) promotes proliferation and differentiation. How was differentiation being measured? It is surprising that DRG neurons can still differentiate. What was it exactly being measured to come up with this conclusion? More information needs to be provided in the Material section.

Neurite length was assessed to demonstrate the effect of NGF and mutants. Based on the images shown in figure 3, with the dense neurites intertwined so much, how was the length of individual neurons being measured?

The NGF and mutants were added to the culture for 3-4 days (in DRGs) and up to 8 days in PC12 cells, it is also surprising a single dose of protein could be functional for that long. Detailed experimental procedure will help solving these confusion.

What is the wavelength of the luminescent product for the TrkA assay?

Authors' Response 20-Dec-2018

Dear Editors-in-Chief Professor Bolam and Professor Foxe and reviewers of the European Journal of Neuroscience,

Thank you for your comments on our manuscript entitled “Identification of amino acid residues of nerve growth factor important for neurite outgrowth in human dorsal root ganglion neurons” (ID: EJN-2018-08-25857). The comments indeed helped us revise and improve the manuscript. We have tried to answer all your questions and changed the manuscript according to your suggestions. The changes in the revised manuscript are underlined and we have also provided an unmarked version.

We would like to express our sincere appreciation to all of you for considering our manuscript for publication after revision. Please do not hesitate to contact us if you have any further questions or comments.

EJN European Journal of Neuroscience FENS Federation of European Neuroscience Societies

Kind regards,
Pontus Forsell, PhD
On behalf of all authors

AlzeCure

1. *There are too many abbreviations in abstract for it to be accessible to the general readership of EJN.*

   Thank you for noting that the number of abbreviations made the abstract difficult to read. The number of abbreviations in the abstract has now been reduced and the abbreviations still used are clearly explained. The abbreviations now used in the abstract are words that we assume researchers commonly use in database searches and these abbreviations can therefore contribute to increased number of hits.

2. *The figures need to be of a higher resolution for publication.*

   High resolution TIFF-files are submitted for each figure.

3. *In accordance with EJN policy, please replace bar charts with more informative scatter plots or similar.*

   We of course want to adhere to the EJN policy and have therefore removed the bar chart. The western blot figures are now present in their native form and are described in the text and in the figure legend.

4. *Please include a graphical abstract with text.*

   A graphical abstract text and a graphical abstract figure are now submitted.

5. *Ensure that the reporting of statistical data adheres to EJN guidelines, notably report precise P values.*

   Statistical data is now in line with EJN guidelines and precise P values are reported, except when the P value is less than 0.0001.

6. *Include direction and magnitude of changes/differences in the text as well as the statistical data.*

EJN European Journal of Neuroscience FENS Federation of European Neuroscience Societies

The suggestion regarding direction or magnitude of change is good and we have clarified the text accordingly. The text and the statistical data are now supplemented with direction and magnitude of change/difference. For example in section 3.1, “The maximum neurite length per neuron of NGF-K95A/Q96A was approximately twice that of wild-type NGF in human fetal DRG neurons at 1.5 µg/ml.” or further down in the same section “The NGF-R100E mutant was the most potent of the three tested NGF mutants in human fetal DRG neurons, even more potent than wild-type NGF, in terms of proliferation ($p = 0.0495$) and differentiation ($p = 0.0339$).

7. *Typo '...incubated with different amounts of wild-type NGF or NGF...'*

Unprecise formulations are now better defined. For example, in section 3.1 “In order to study the effects of NGF mutants on proliferation and differentiation, human fetal DRG neurons were incubated for 4 days with increasing concentrations of wild-type NGF or NGF mutants ranging from 1 pg/ml to 100 ng/ml (Fig. 2).”.

8. *When ‘cell number’ is referred to in the figures indicate what this means. There is no description of cell counting in the methods except to say that it was performed. Similarly, as pointed out by the reviewer, much more detail about measurement of neurite lengths.*

Thank you for pointing out that cell counting methods should be described in more details. We have added additional information of cell counting in *Material and Methods* as exemplified in section 2.3, on page 7, line 3. In the manuscript we now describe that before seeding, cells were counted using Trypan blue solution and a Bürker chamber. Numbers of nuclei in immunocytochemistry analysis were counted using Hoechst nuclear stain and cells were defined as β-tubulin positive objects with nucleus and cell soma. Immunocytochemistry quantifications are exemplified in section 2.5, on page 8.
For details about measurement of neurite length, see paragraph 13 below.

9. *It is not transparent as to how many experiments/replications were performed. For example, in Fig 4: 'Each point represents mean ± SEM (n = 4) from one representative experiment.'; it is not clear what this means, why only a representative example is reported or how we know it is representative. Figure 6 is even more confusing 'Each point represents mean ± SEM (n = 4) from at least four independent experiments'.*

We agree that the number of replicates used for different experiments were not clearly stated. We have addressed the reviewer’s comments regarding number of replicates by clarifying the number of replicates, as exemplified in figure caption number 4: “Seven independent biological repeats with four technical replicates were conducted”. In figure 6A, the experiment was repeated five times with four technical replicates each time and in figure 6B the experiment was repeated four times with four technical replicates each time. Our intention with the former figure caption was to express that the experiments in figure 6 were repeated at least four times with four technical replicates each time.

10. *In general, the figure legends need to better describe the figures.*

The reviewer's comment is fair, and we have extended the figure legends to better describe the figure and the methodology.

11. *Most of the findings were reported in graphs (Fig. 2,4,5,6,7), and except figure 2 where the cell images were then shown in figure 3, most of the other figures did not have accompanying images to support the quantification. This is particularly important when intensity measurement was performed on the neurons, such as fig 7.*

    We agree that several figures were not accompanied by representative images of the cells. Thus, we have included high-content images for figure 5 and 7. In figure 5, showing Hoechst and β-tubulin-stained images of PC12 cells, and in figure 7, showing images of phospho-ERK1/2 staining in DRG. Vehicle vs wild-type NGF treated cells are shown to demonstrate the immunocytochemistry high-content images. The results presented in Figure 6 is not based on image analysis and we have therefore not included any additional images for figure 6.
    In the Addendum in this letter and in the Graphical Abstract Figure, we have included four high-content images that show β-tubulin positive neurite outgrowth and pERK1/2 staining in DRG neurons in relation to vehicle, wild-type NGF or NGF-R100E treatment at 1 ng/ml or 100 ng/ml. With these images we like to exemplify the pronounced difference between 1 ng/ml wild-type NGF and 1 ng/ml NGF-R100E treated DRG neurons, both regarding neurite outgrowth and pERK1/2 staining. In the graphs E and F in the Addendum, these conditions are found at log concentration -9 (g/ml), wild-type NGF (black circles) and NGF-R100E (red squares).

12. *In Figure 2 and 3 where DRG were used for the experiments, the authors claimed that the NGF (wildtype and some mutant) promotes proliferation and differentiation. How was differentiation being measured? It is surprising that DRG neurons can still differentiate. What was it exactly being measured to come up with this conclusion? More information needs to be provided in the Material section.*

    We believe that the fact that the DRGs are of embryonal origin makes them more likely than adult DRGs to differentiate upon stimulation with growth factors such as NGF. Differentiation of DRG neurons was defined as the transition from a rounded cell with nucleus to a β-tubulin positive neuronal cell with cell soma and expansive neurites.
    We have added additional information under *Materials and methods*, section 2.5, page 7-8, to describe differentiation and high-content imaging analysis. We hope

that this additional information will clarify the procedure. Analytical images are also included in figure 3 to demonstrate the automated high-content imaging analysis.

13. *Neurite length was assessed to demonstrate the effect of NGF and mutants. Based on the images shown in figure 3, with the dense neurites intertwined so much, how was the length of individual neurons being measured?*

    This is a very good question and we would like to refer to the benefits with automated high-content imaging analysis.
    The length of each neurite was measured using an algorithm in the analysis software, and as such, the measurement of the neurites is unbiased. We have included an example where the image in figure 3 is accompanied with an image showing the mask that was obtained after algorithm-identified neurites. This has also been clarified in *Materials and methods,* in section 2.5. The average length per neurite was quantified from the neurite total length divided by number of selected cells in the field of view. Four fields of view per well were measured, and the average neurite length in µm per neuron per well is reported.
    Comparing similar treated cell cultures will also minimize any bias in the assay, as all analyzes are performed with the same algorithm.

14. *The NGF and mutants were added to the culture for 3-4 days (in DRGs) and up to 8 days in PC12 cells, it is also surprising a single dose of protein could be functional for that long. Detailed experimental procedure will help solving these confusions.*

    We agree that it is surprising that a single dose of NGF can be active for such a long time, but it is not an unusual procedure as judged by published work by others in the field. However, to address the stability of the proteins, a stability test has been performed with wild-type NGF and the NGF mutants. Wild-type NGF or mutant NGF was pre-incubated for 3 days in assay medium at +37ºC and then added to the PC12 cells and incubated for another 4 days. No difference in proliferation or differentiation parameters was found between PC12 cells treated with pre-incubated or freshly prepared wild-type NGF or mutant NGF after 4 days in culture. These data are not mentioned in the manuscript since we believe that the data is outside the scope of the manuscript.

15. *What is the wavelength of the luminescent product for the TrkA assay?*

    Thank you for raising this essential issue.
    Luminescence readout collects signal from all wavelengths. Some instrument or kit manufacturers may suggest a cutoff filter at high wavelengths, but no wavelength setting is needed for luminescence readout when using the reagents from DiscoverX. Thus, we collected luminescence from all wavelengths.

EJN European Journal of Neuroscience
FENS Federation of European Neuroscience Societies

Addendum

We would like to display some images of human fetal DRG neurons and a few selected conditions of both neurite outgrowth and pERK1/2 staining in relation to wild-type NGF or NGF-R100E treatment, to highlight the potency difference between the two proteins.

A B C D

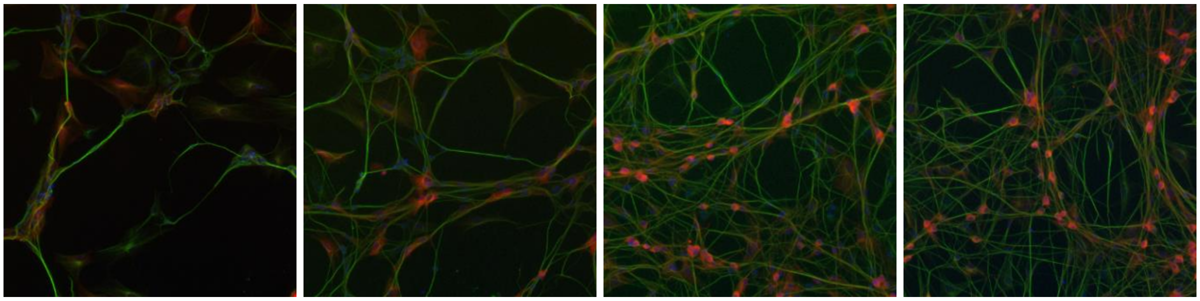

E F

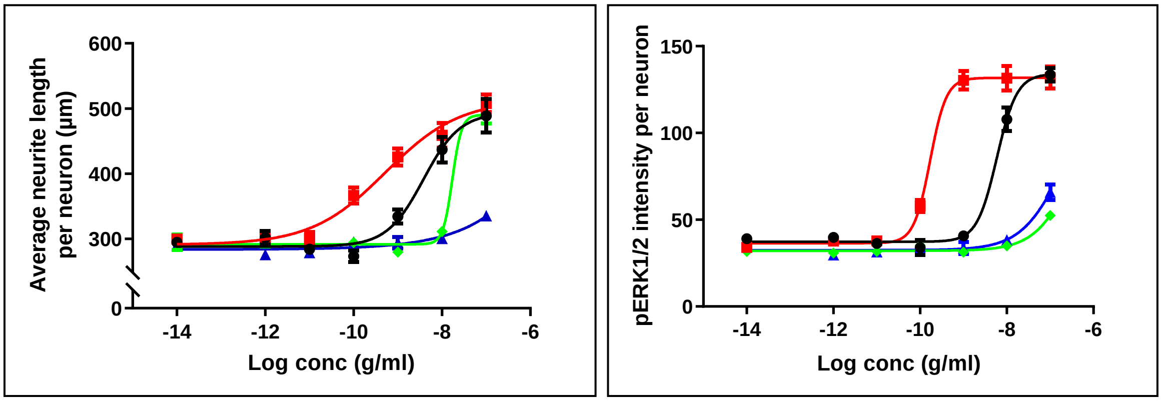

Representative high-content images of human fetal dorsal root ganglion neurons treated with (A) vehicle, (B) 1 ng/ml wild-type NGF, (C) 1 ng/ml NGF-R100E, and (D) 100 ng/ml wild-type NGF, Hoechst nuclear stain (blue), anti-β-tubulin (green) and anti-pERK1/2 (red). Dose-response curves with wild-type NGF or NGF mutants ranging from 1 pg/ml to 100 ng/ml for 4 days, mean ± SEM. Differentiation (defined as average neurite length per neuron in µm) of human DRG neurons is shown in figure E and ERK1/2 phosphorylation (average pERK1/2 staining intensity per neuron) is shown in figure F, after a 4-days treatment with wild-type NGF (black circles), NGF-R100E (red squares), NGF-W99A (green diamonds) or NGF-K95A/Q96A (blue triangles).

EJN European Journal of Neuroscience FENS Federation of European Neuroscience Societies

2nd Editorial Decision 04-Apr-2019

Dear Dr. Forsell,

First of all, apologies for the inordinate length of time it has taken to deal with your re-submitted manuscript. We had difficulty engaging reviewers and in view of the time we have decided to go ahead with a single reviewer.

As you can see below, the reviewer considers your paper to be interesting and of value but raises a few issues concerning the quality control in the preparation of NGF proteins and quantification of data that will need to be addressed in a revised version before we can further consider it for publication in EJN. Please carefully respond to each point that he/she has raised in the revised version.

When revising the manuscript, please embolden or underline major changes to the text so they are easily identifiable and please don't leave 'track change' formatting marks in your paper. Please ensure that you provide a text and a figure file for the Graphical Abstract (as detailed in the instructions below). When carrying out your revisions please refer to the checklist below and visit the EJN author guidelines at www.ejneuroscience.org

When finalized, please upload your complete revised manuscript onto the website, as a Word file (.doc, or .docx). Please also ensure that a complete set of tables and figures is included as separate files, even if these have not changed from the originals. At this stage it is necessary to provide high resolution figures. Please see important instructions below.

Please go into https://mc.manuscriptcentral.com/ejn - Author Centre - manuscripts with decisions where you will find a 'create a revision' link under 'actions'. We ask that you please indicate the way in which you have responded to the points raised by the Editors and Reviewers in a letter. Please upload this response letter as a separate Word (.doc or PDF) file using the file designation "Authors' Response to Reviewers" when uploading your manuscript files. Please DO NOT submit your revised manuscript as a new one. Also, please note that only the Author who submitted the original version of the manuscript should submit a revised version.

If you are able to respond fully to the points raised, we would be pleased to receive a revision of your paper within 12 weeks.

Thank you for submitting your work to EJN and apologies again for the long delay.

Best wishes,

Paul Bolam & John Foxe
co-Editors in Chief, EJN

Reviews:

Reviewer: 2

EJN European Journal of Neuroscience FENS Federation of European Neuroscience Societies

Comments to the Author
The key point of the manuscript is that the R100E NGF mutant was more potent than its wildtype counterpart in activating TrkA, pErk. If vigorously validated, this would be interesting to the field of NGF. However, there are significant deficiencies in the data presented in the manuscript: 1) there was no quality control in the preparation of NGF R100E and other NGF proteins, at minimum, a silver staining gel of the different protein preps needs to be shown; 2) there was no quantification of pTrkA; 3) the levels of pErk need to be quantitated as well in Fig 8 A, B, C.

Additionally, what is the justification of using DRG neurons from aborted human fetus? In my own opinion, PC12 cells and primary DRG neurons from mouse and rat should be sufficient for testing the various NGF mutants. there is really no need for this.

Authors' Response 27-Jun-2019

Dear Editors-in-Chief Professor Bolam and Professor Foxe and reviewers of the European Journal of Neuroscience,

We are pleased that our manuscript “Identification of amino acid residues of nerve growth factor important for neurite outgrowth in human dorsal root ganglion neurons” (ID: EJN-2018-08-26087) will be considered for publication after revision. The reviewer had comments and suggestions which have improved the revised version of the manuscript. We have performed the experiments that the reviewer asked for and tried to answer the reviewer’s questions, to the best of our ability. The changes in the revised manuscript are underlined and we have also provided an unmarked version.
Your insights were truly helpful. Please do not hesitate to contact us if you have any further questions or comments.

Kind regards,
Pontus Forsell, PhD
On behalf of all authors

AlzeCure

EJN European Journal of Neuroscience FENS Federation of European Neuroscience Societies

1. *There was no quality control in the preparation of NGF R100E and other NGF proteins, at minimum, a silver staining gel of the different protein preps needs to be shown.*

   After the final purification process of the proteins, the proteins were found >95% pure as judged by SDS-PAGE gel, as stated in our previously submitted manuscript. The qualities of the proteins were not clearly demonstrated, and we agree with the reviewer that a protein staining of the protein preparations must be presented. We therefore include data and results for both silver staining and Coomassie blue staining of the wild-type NGF and the three mutants, please see figure 1B and 1C and section 2.2 in Material and methods. These protein stains demonstrate >95% pure proteins, with the correct size (13 kDa) and equal concentration.

2. *There was no quantification of pTrkA. The levels of pErk need to be quantitated as well in Fig 8 A, B, C.*

   We thank the reviewer for his/her suggestion to improve or study with quantification of pTrkA and pERK1/2. To address direct quantification of pTrkA and pERK1/2 we decided to perform a solid phase sandwich enzyme-linked immunosorbent assay (ELISA) from R&D Systems. We have performed western blots to identify the right size of the proteins with pTrkA and pERK1/2 antibodies. Both ELISA and western blot are immune-based assays, but ELISA is more sensitive than western blot. The use of ELISA allowed us to quantify pTrkA and pERK1/2 levels with high precision.

   Phospho-TrkA

   The result of the ELISA analysis of pTrkA from three independent biological repeats of U2OS-TrkA cells is now added to figure 6 as figure 6 C. The figure legend to figure 6 is changed accordingly. The results from the phospho-TrkA ELISA assay is added in the result section in the latter part of section 3.3. A description of the procedure has been added to the new material and method section 2.7.

   The data showed that the NGF-R100E mutant is significantly more effective regarding phosphorylation of the TrkA receptor, compared with wild-type NGF. The amount of phosphorylated TrkA is around 3 times more for NGF-R100E than for wild-type NGF at all concentrations from 10-300 ng/ml. The ELISA analysis also verified that the ability to phosphorylate TrkA of the other two tested NGF mutants, W99A and K95A/Q96A, is significantly reduced.

   Phospho-ERK1/2

   Western blot analysis of pERK1/2 levels from three existing independent biological repeats of DRG neurons have now been quantified and the results are added and

shown in figure 8B. In our previously submitted version we showed a significantly more potent activation of pERK1/2 in the immunocytochemistry analysis of pERK1/2 and DRG neurons (Fig. 7C) while the western blot analysis at 30 ng/ml NGF or mutant NGF only showed increased levels of pERK1/2 for NGF-R100E compared with wild-type NGF. Phospho-ERK1/2 levels for W99A and K95A/Q96A were decreased, but not significantly using western blot (Figure 8B).

To better monitor the modest levels of phospho-ERK1/2 in U2OS-TrkA cells and PC12 cells, phospho-ERK1/2 ELISA analysis was performed.

ELISA analysis of pERK1/2 from four independent biological repeats of U2OS-TrkA cells or PC12 cells is shown in the new figures 9A and 9B. In U2OS-TrkA cells, the amount of pERK1/2 is significantly induced after treatment with 100 ng/ml NGF-R100E compared with wild-type NGF. The result is described in section 3.4. The procedure is described in the new material and method section 2.7, along with the phospho-TrkA ELISA procedure.

3. *What is the justification of using DRG neurons from aborted human fetus? In my own opinion, PC12 cells and primary DRG neurons from mouse and rat should be sufficient for testing the various NGF mutants, there is really no need for this.*

We agree with the reviewer that a justification of choice of cells is warranted and have added a paragraph in the discussion about this relevant issue (page 16, line 8-14).

Regarding the choice of cells to study, DRG neurons from aborted fetuses is a source of human neurons dependent on NGF for survival and differentiation. According to us, human DRG neurons is a suitable cellular system in evaluating NGF mutants in the process of understanding neuronal survival and differentiation in man. Small structural changes in NGF such as the R100E or the W99A mutations lead to significant changes in cellular responses in our cellular models, which are mediated by TrkA. Since rat TrkA and human TrkA only are 86% identical in their primary amino acid sequence, with the majority of differences being found in the extracellular domain, we think that the use of human TrkA or human cells expressing endogenous TrkA can be of importance to obtain a correct biological response, especially when using human NGF or human NGF-mutants. There are indeed differences between PC12 cells and human DRG neurons and how these cell types respond to human NGF-R100E and NGF-W99A as indicated in figures 2, 4, 5, 7, 8 and 9. Identifying the mechanisms behind the differences between the responses of rat and human cells is beyond the scope of this manuscript.
